# A comparison of late gadolinium enhancement magnetic resonance imaging and left atrial endocardial voltage

**DOI:** 10.1186/1532-429X-15-S1-P67

**Published:** 2013-01-30

**Authors:** James Harrison, Nick Linton, Steven Williams, Rashed Karim, Kawal Rhode, Matthew Wright, Tobias Schaeffter, Reza Razavi, Mark O'Neill

**Affiliations:** 1 Division of Imaging Sciences & Biomedical Engineering, King's College London, London, UK; 2 Department of Cardiology, St Thomas' Hospital, London, UK

## Background

Following catheter ablation for atrial fibrillation (AF), late gadolinium enhancement magnetic resonance imaging (LGE MRI) may be able to visualise areas of fibrosis and therefore reduced endocardial voltage. Unlike previous studies describing qualitative visual comparisons, we have developed a new quantitative technique for comparison of left atrial (LA) endocardial voltage with 3D LGE MRI signal intensity and have applied it in patients undergoing repeat left atrial ablation.

## Methods

Ten patients who had undergone previous catheter ablation for AF, and who represented with either paroxysmal AF (n=4) or atrial tachycardia (AT) (n=6) underwent pre-ablation LGE MRI. A 3D LA reconstruction was created by projecting the LGE data on to a segmented LA shell (signal intensities were displayed as the number of standard deviations (SD) from the mean signal intensity of the atrial blood pool).

During the ablation procedure, high density (mean number of points: 368 ± 127) LA endocardial voltage maps were acquired in either sinus rhythm or AT using CARTO 3 (Biosense Webster).

The 3D LGE MRI reconstructions and endocardial voltage maps were manually segmented into LA regions (left and right WACA, roof, mitral line, anterior, posterior, inferior, septum and lateral) using custom-written software to derive the mean signal intensity and endocardial voltage for each segment.

## Results

Mean LGE MRI signal intensity and endocardial voltage were calculated for a total of 131 atrial segments in the ten patients.

In the atrial segments in which the mean LGE MRI signal intensity was low (0 to 3 SD from the mean intensity of the atrial blood pool - ‘healthy'), the mean ± SD endocardial voltage was 0.86 ± 0.7 mV, whereas when the mean LGE MRI signal intensity was high (>3 SD - ‘scar'), the mean endocardial voltage was significantly lower at 0.51 ± 0.4 mV (p<0.004) (Figure).

**Figure 1:**
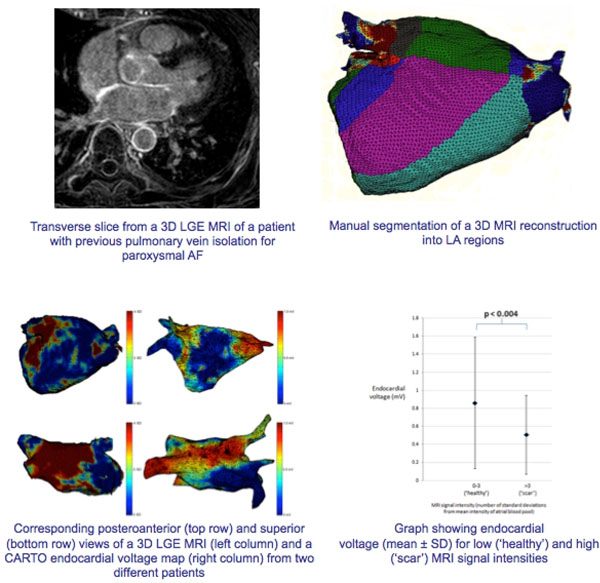


## Conclusions

High LGE MRI signal intensity correlates with areas of low endocardial voltage, however, low voltage can still occur in areas of low signal intensity on LGE MRI. Further refinement of this technique is needed to permit the non-invasive assessment of atrial substrate prior to repeat catheter ablation.

## Funding

British Heart Foundation Clinical Research Training Fellowship.

